# The Effect of Early Applied Robot-Assisted Physiotherapy on Functional Independence Measure Score in Post-Myocardial Infarction Patients

**DOI:** 10.3390/healthcare10050937

**Published:** 2022-05-18

**Authors:** Peter Bartík, Michal Vostrý, Zuzana Hudáková, Peter Šagát, Anna Lesňáková, Andrej Dukát

**Affiliations:** 1 Health and Physical Education Department, Prince Sultan University, Riyadh 12435, Saudi Arabia; sagat@psu.edu.sa; 2 Faculty of Education, J. E. Purkyně University, 40096 Ústí nad Labem, Czech Republic; michal.vostry@ujep.cz; 3 Centre for Social Innovation and Inclusion in Education, J. E. Purkyně University, 40096 Ústí nad Labem, Czech Republic; 4 Faculty of Medical Studies, J. E. Purkyně University, 40096 Ústí nad Labem, Czech Republic; 5 Faculty of Health, Catholic University, 034 01 Ružomberok, Slovakia; zuzana.hudakova@ku.sk (Z.H.); anna.lesnakova@ku.sk (A.L.); 6 Department of Health Care Studies, College of Polytechnics, 58601 Jihlava, Czech Republic; 7 SNP Central Military Hospital, Faculty Hospital, 034 01 Ružomberok, Slovakia; 8 Fifth Department of Internal Medicine, Comenius University in Bratislava, 814 99 Bratislava, Slovakia; andrej.dukat@fmed.uniba.sk

**Keywords:** robot-assisted therapy, FIM score, myocardial infarction, first phase cardiac physiotherapy

## Abstract

Robot-assisted training has been widely used in rehabilitation programs, but no significant clinical evidence about its use in productive working-age cardiac patients was demonstrated. Thus, we hypothesized that early applied robot-assisted physiotherapy might provide additional treatment benefits in the rehabilitation of post-myocardial infarction (MI) patients. A total of 92 (50 men, 42 women) hospitalized post-MI patients with the age of 60.9 ± 2.32 participated in the research. An early intensive physiotherapy program (7×/week, 2×/day) was applied for each patient with an average time of 45 min per session. Patients were consecutively assigned to Experimental group (EG) and Control group (CG). Then, 20 min of robot-assisted training by Motomed letto 2 or Thera-Trainer tigo was included in all EG physiotherapy sessions. The Functional Independence Measures (FIM) score at the admission and after 14 days of rehabilitation was used for an assessment. When analyzing time * group effect by repeated-measures ANOVA, we reported that EG showed a higher effect in ADL (*p* = 0.00), and Motor indicators (*p* = 0.00). There was no statistically significant effect reported in the Social indicator (*p* = 0.35). Early rehabilitation programs for post-MI patients might be enhanced by robotic tools, such as THERA-Trainer tigo, and Motomed letto 2. The improvement was particularly noticeable in mobility and ADLs.

## 1. Introduction

Coronary heart disease (CHD) is the leading cause of morbidity and mortality worldwide. The most common form of CHD is myocardial infarction [1]. The number of post-MI patients hospitalized is increasing gradually. The basic therapeutic principles for the treatment of this disease are the same for all age groups. However, it is necessary to modify the treatment and subsequent therapy with respect to the patient’s age [2]. Age is one of the decisive factors that has a major impact on the therapeutical process [3]. Other factors include gender and patients’ individual needs. The therapy prescribed should take all these factors into account [4]. The incidence of MI increases with age. In the United States, people over 65 years represent 13% of the total population, but they form almost half of all hospitalized patients with this diagnosis. The MI prevalence in the population of 40–59 age is 3.3% in men, and 1.8% in women, while in the population of 60–79 age it is 11.3% in men, and 4.2% in women [5]. The incidence in the population of the Czech Republic is very similar, with the highest incidence rate in men and women in the 70 to 79 years age group [6]. In South Asians, the incidence is even higher in the most age groups. The incidence rate ratio is 1.45 for South Asian compared to non-South Asian men and 1.80 for South Asian women [7]. Based on the above stated, it is also important to note that elderly patients have a higher incidence of co-morbidities which may contribute to higher mortality [8]. In younger MI patients, positive family history is often present, while older patients suffer from hypertension, diabetes mellitus, or obesity more frequently [9]. The myocardial infarction is often atypical in diabetic patients. It is without common stenocardia, which is the reason why such patients seek medical attention only after developed complications have manifested [10].

The number of hospitalized post-MI patients is extensive, so it is a demand for effective rehabilitation. The target group in our study were patients of the active working age (<64 years) with a need for an effective physiotherapy process to be able to get back to the working process and daily life as soon as possible. The rehabilitation process of cardiac patients is divided into four phases. The first phase is focused on hospital rehabilitation and was the period of our investigation. Its main goal is to prevent deconditioning, thromboembolic complications, and to prepare the patient for discharge and return to normal daily life as soon as possible. The second phase is focused on immediate post-hospital rehabilitation. The duration is about three months and the focus is mainly on lifestyle change and adherence to secondary prevention. The third phase is a period of stabilization, in which the emphasis is on regular endurance training and consolidating changes in a healthy lifestyle. The last fourth phase is then focused on maintaining the status quo, which means regular, long-term compliance with said principles [11,12]. Despite the availability of the presented therapeutic options nowadays, it is important to mention the prognosis of the disease itself. It is worse in older patients than in the younger population. Improving the rehabilitation care of patients with myocardial infarction leads to a prolongation of their lives [13].

The current modern approach in inpatient rehabilitation is focused on robot-assisted therapy with regard to the body weight of patients which might be a limitation for rehabilitation procedures [14]. Robotic devices in rehabilitation we can see in use today are continuously under intensive development to improve their effectiveness in physiotherapy programs. In the case of traditional concepts of physiotherapy, it is directed mainly to achieving functional improvement of motor or cognitive abilities [15]. In the case of cardiac patients, robot-assisted training has been widely used in rehabilitation programs, but no significant clinical evidence to use it in productive working-age cardiac patients was demonstrated. Thus, the main contribution of this study is to provide the evidence supporting the usage of robotic tools in early rehabilitation stage of post-MI patients. We hypothesized that early applied robot-assisted physiotherapy might provide additional treatment benefits in the rehabilitation of post-MI patients. The purpose of the study was to investigate what is the effectiveness of robot-assisted physiotherapy in early stage post-MI patients of productive working age.

## 2. Materials and Methods

### 2.1. Subjects and Experimental Setup

The research sample consisted of 92 participants of the productive working-age 60.9 ± 2.32 (55–64 years), with a BMI of 32.2 ± 4.84, representing 50 men and 42 women. Data collection took place in the Department of cardiology, Masaryk Hospital, Ústí nad Labem, Czech Republic. All patients included in the study were hospitalized due to the myocardial infarction (MI), ICD codes—10: I21. The secondary diagnosis was obesity or diabetes mellitus, or a combination of both. All underwent early mobilization within the 2nd–3rd day of hospitalization after approval from a cardiologist. These patients were sufficiently stable to start cardiac rehabilitation based on the following conditions: stable blood pressure, stable heart rate (HR), no angina pectoris, no shortness of breath, Ejection fraction > 0.45, no resting or stress ischemia, and no arrhythmia. The following parameters were regularly assessed when in the physiotherapy sessions: Borg score below 13 (6–20), resting HR increased max + 20 bpm, HR below 120 bpm, exercise up to tolerance if non-symptomatic. If any parameter was not met, the training was completed only supine on the bed, or not at all if the symptoms persisted. Patients with complications were excluded from the research.

**Figure 1:**
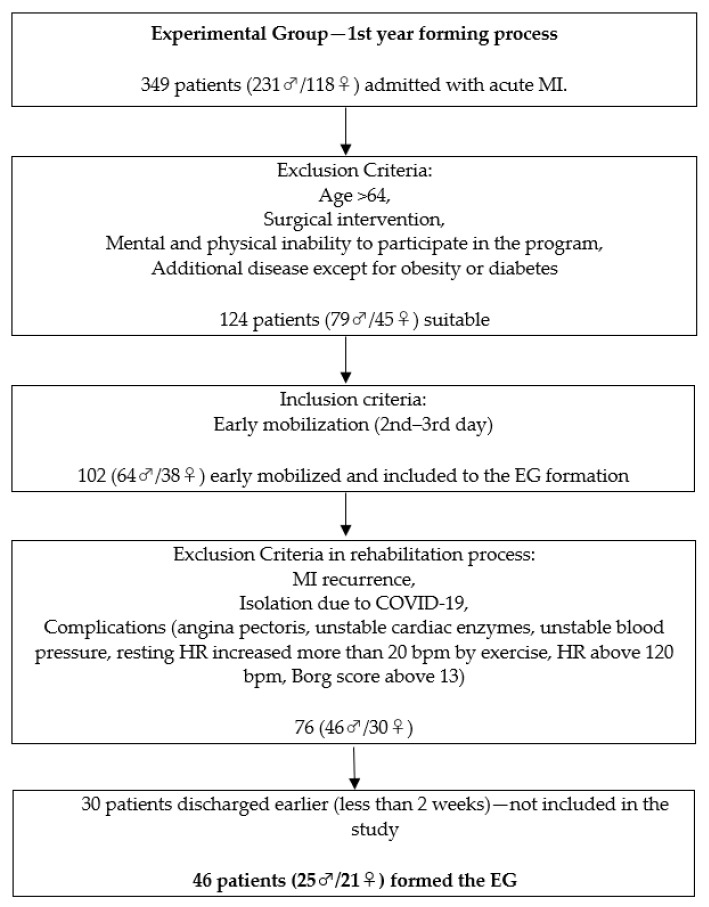

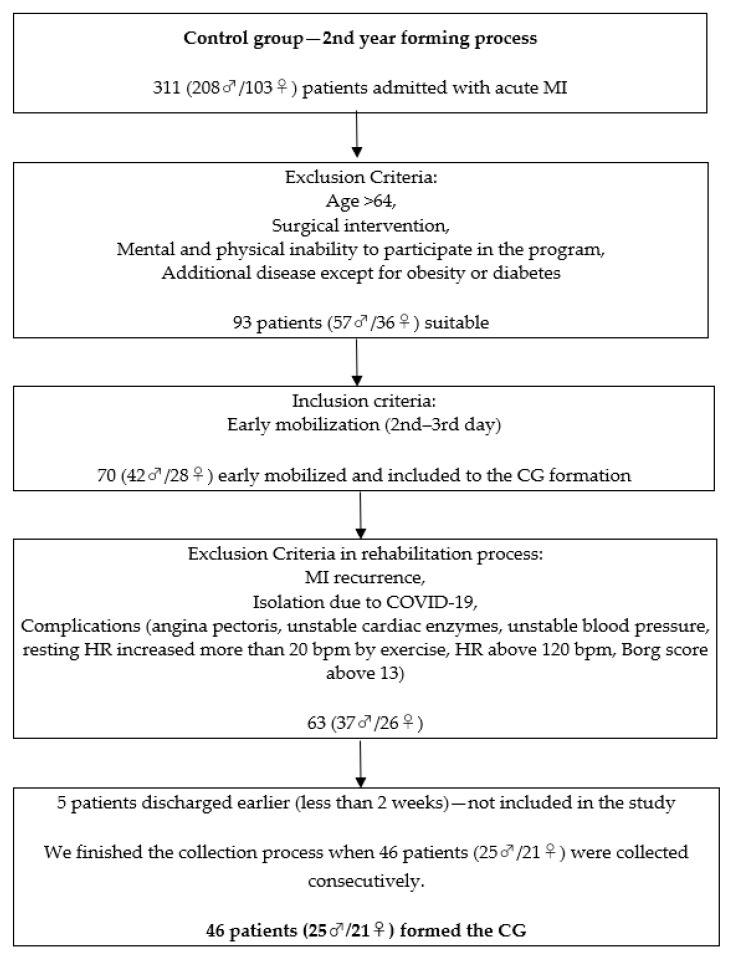
Research flowchart.

All patients had to pass the inclusion criteria. Data collection was performed consecutively. The first 46 patients (25 ♂/21 ♀) that passed the inclusion criteria formed the EG and the second 46 patients formed the CG. The original target of EG was a minimum of 50 (25 ♂/25 ♀). However, we reduced it by four on the female side due to the inability to recruit an appropriate number till the end of the time period devoted to the EG collection (one year). Afterward, the CG was formed (25 ♂/21 ♀) with the same inclusion criteria. The timeframe of the study lasted two years. The distribution of the subjects based on gender was equal in both research groups. We outline the whole process in the research flowchart (Figure 1). All patients that participated in the experiment were explained the research details before they signed the written consent. The participants were blinded to the research hypothesis.

Inclusion criteria:

- Individuals after MI, ICD codes—10: I21;
- Age < 64;
- At least 1 days of physiotherapy training before discharge;
- Mental and physical ability to participate in the program;
- No active angina pectoris, stable cardiac enzymes, stable blood pressure, pulse, and respiratory rate within a range that allowed the patient to exercise;
- Early mobilization (2nd–3rd day of hospitalization);
- No surgical intervention (catheterization not included).
- Exclusion criteria:
- Early discharge from the unit (less than 14 days of physiotherapy program);
- Complicated recovery;
- MI recurrence;
- Late mobilization (more than third day);
- Additional disease except for obesity or diabetes;
- Isolation due to COVID-19.

### 2.2. Intervention

Both EG and CG participants started an early intensive physiotherapy program seven times a week (two times a day) with an average time of 45 min per session. The early program for patients after MI was focused on preventing decondition and thromboembolic complications, improving adaptation to physical activity, and preparing patients to return to ADLs. The early physiotherapy program was performed by four physiotherapists experienced in the field. The motivation factor was the same for both research groups since the study was blinded to the hospital staff. The type and intensity of exercise and the position (standing, sitting, lying in a supine position) depended on the patient’s condition. Exercises in both EG and CG were the same except for the robotic intervention. The majority of movements performed during the physiotherapy sessions had repetitive analytical character, meaning they had the same range of motion and direction while it was not based on real-world situational biomechanics, such as functional movements. The movements were repetitively performed with specific phases and rhythm equal for both EG and CG.

Physiotherapy units for EG included active-assisted and active repetitive analytical movements of the upper and lower limbs lying on the bed (5 min), active exercise of repetitive analytical movements in a sitting position (5 min), mobilization to a standing position, and a short walk, active exercise of repetitive analytical movements in a standing position, and a short walk up and down the stairs (15 min). Then, 20 min of robot-assisted training with repetitive movements was implemented in all EG physiotherapy sessions. Both legs and arms were evenly involved in the training on a regular basis. Robotic device applications in EG always came with the start of the mobilization and rehabilitation program.

Physiotherapy units for CG included active-assisted and active repetitive analytical movements of the upper and lower limbs lying on the bed (10 min), active exercise of repetitive analytical movements in a sitting position (10 min), mobilization to a standing position, and a short walk, active exercise of repetitive analytical movements in a standing position, and a short walk up and down the stairs (25 min).

We applied the following devices for the early-stage rehabilitation program in EG: MOTOmed letto 2 (RECK-Technik GmbH & Co. KG, Betzenweiler, Germany), and THERA-Trainer tigo (Medizintechnik GmbH, Hochdorf, Germany).

MOTOmed letto 2 is a motor-assisted bed model training device with an automatic system for either legs or arms mobilization and training in a supine or sitting position often used for bed-ridden patients. THERA-Trainer tigo is a motor-assisted training device with an automatic system for either legs or arms mobilization, and training in a sitting position. Both devices allow passive, active-assisted, active, or active against resistance movements. The advantage of both is the possibility of application with the function of presetting and memory of training regime level. It is safe to let patients work out without active supervision. The presence of a physiotherapist is needed only at the beginning and the end of the training unit and when adjusting between arms and legs program. The position (supine or sitting), and the level of resistance for each EG patient was based on the actual condition. The training process had a tendency to increase the difficulty level of each following session, while the time was fixed from the beginning.

Both devices are regularly used in rehabilitation programs in our department, and their application is justified by several studies. Following authors reported a positive effect of MOTOMED letto 2 in the early physiotherapeutic intervention [16,17], while the positive effect of THERA-Trainer tigo was declared by the following researchers [18,19].

### 2.3. Assessment

The Functional Independence Measure (FIM) score is the standardized tool to evaluate the patient’s functional level and independence on admission and discharge from the hospital. FIM score at the admission and after 14 days of rehabilitation (28 sessions) was used for an assessment of the subjects. Three indicators of FIM were evaluated individually—ADL, Motor, and Social. Standardized FIM record sheets in the Czech language were used.

All FIM scores were evaluated by four experienced physiotherapists working in the department. Both input and output assessments of the particular patient were always performed by the same physiotherapist. All physiotherapists included in the assessment and therapy process were blinded to the study details. We concluded the inter-rater reliability sufficient since it was performed by four trained and experienced physiotherapists and it is supported by scientific literature [20]. Orders for the specific physiotherapy interventions for CG and EG came only from the researcher working in the department after consultation with the cardiologist. The same researcher was regularly supervising the experiment process. However, he did not interfere with the FIM evaluation process.

Each item of the FIM (total score 18–126) is assessed by 1–7 points. Three research indicators are as follows:

ADL (score 8–56) includes eight items: eating, grooming, bathing, dressing the upper body, dressing lower body, toileting, bladder management, and bowel management;

MOTOR (score 5–35) includes five items: transfers—bed/chair/wheelchair, transfers—toilet, transfers—bath/shower, walk/wheelchair, stairs;

SOCIAL (score 5–35) includes five items: comprehension, expression, social interaction, problem solving, and memory [21].

### 2.4. Sample Size and Statistical Analysis

The sample size calculation was analyzed by the G*Power software version 3.1.9.4. The interval of confidence was set to 95%, the margin of error to 5%, and the probability of success to 0.5. It was determined that the minimum total sample size that should participate in the study to have a representative sample of the studied population was 54.

All data were analyzed using IBM SPSS version 25. Statistical analysis included a descriptive analysis of general characteristics by using the mean and standard deviation. Gender independence was analyzed by the Chi-square test. Normality of dataset distribution was analyzed by Kolmogorov–Smirnov and Shapiro–Wilk tests. The Paired T-test was used for the analysis of the FIM indicators differences. The Mann–Whitney U test was used for age, BMI, diabetes type II, Borg score, and HR differences comparisons. The time * group effect was analyzed using Repeated Measures ANOVA, taking the group as between factor and time as within factor. To estimate the effect size (ES), after applying the T-test in FIM indicators, the following formula was used: ES = (X_1_ − X_2_)/√(S_1_^2^ + S_2_^2^)/2. An ES of 0.2 was considered small, 0.5 moderate, and 0.8 large. The statistical significance threshold was set to 0.05.

## 3. Results

Data from 92 participants (50 male/42 female) of the productive working-age 60.9 ± 2.32 (55–64 years), with a BMI of 32.2 ± 4.84, were collected and included in the study. Table 1 reports the demographic characteristics, type II diabetes duration (years), maximum Borg score, resting HR, and maximum HR in effort of all participants, and between research groups differences. Borg score and HR data were obtained during the first day of the physiotherapy program and after one week. No statistically significant differences were reported between CG and EG. Table 2 reports FIM differences between the Admission and 14 days of rehabilitation, with the effect size (Cohen’s d using pooled variance). Table 3 reports time * group analyses of three FIM indicators.

The research brought the following findings. Standardized FIM Scores for both research groups at the admission ranged from Level 4 (Moderate assistance 72–89) to Level 5 (Supervision needed 90–107), and after 14 days of physiotherapy program from Level 5 to Level 6 (Modified independence 108–119). Patients of both groups improved significantly in two weeks in all three FIM indicators—ADL, Motor, and Social (*p* < 0.05).

When analyzing the time * group effect, we reported a statistically significant difference in the FIM-ADL indicator (*p* = 0.00), and the FIM-MOTOR indicator (*p* = 0.00). The effect of the therapy was higher in EG, where the robot-assisted intervention was included in the physiotherapy program. We did not report any statistically significant difference between groups in the FIM-SOCIAL indicator (*p* = 0.35).

## 4. Discussion

Our study revealed that early applied robot-assisted physiotherapy provided additional treatment benefits in the rehabilitation of post-MI patients. The Motomed letto 2 and Thera-Trainer tigo were used in our experiment. We reported a significant difference when analyzing the time * group effect of EG and CG by FIM results, particularly the FIM-ADL indicator, and the FIM-MOTOR indicator, while in the case of the FIM-SOCIAL indicator, we did not report any significant effect of the experimental therapy when time * group effect was evaluated. The presented results indicate an improvement in performing activities of daily living and mobility. The research group of patients improved mainly in the areas of verticalization, hygiene, and mobility. Taking into account an improvement in the monitored areas of the selected patients after two weeks of intervention, in general, we evaluate the combined robot-assisted therapy in a positive way. The robot-assisted rehabilitation effect is relatively unknown in the professional public when considering post-MI cardiac patients. No research has been published regarding this topic. There are studies confirming the positive effects of robot-assisted physiotherapy in research samples different from working-age post-MI cardiac patients presented in this study [22,23,24,25]. The above-mentioned authors reported additional treatment benefits when robotic physiotherapy was applied, while other studies are relatively skeptical of such claims putting it on the same level as the conventional approach with no extra benefits [26,27,28]. The other study concludes that although robot-assisted therapy can improve the motor skills of individuals, this phenomenon is not completely proven and further research is needed [29]. The presented results report that robot-assisted therapy might have a positive effect and bring additional treatment benefits to patients after myocardial infarction. The FIM indicators scores of the experimental group with robot-assisted physiotherapy intervention improved in ADLs and mobility with a statistically significant difference comparing the group with a casual physiotherapy approach. Based on the results we can recommend using robot-assisted devices in the early rehabilitation plan of post-MI patients. Robot-assisted physiotherapy has a tendency to be widely applied in the field even more in the following years considering the population aging trend which causes a need to adapt to the newly emerging demographic situation. The aim of such adaptation is primarily to prevent the exclusion and discrimination of the older age group where robot-assisted therapy might be very useful and effective. All interventions should lead to an active movement even during aging [2].

In recent years, robotic systems have been playing an increasingly important role in physiotherapy. The aim of these platforms is to aid the recovery process by assisting patients to perform a number of controlled tasks, thus effectively complementing the role of the physiotherapist [30]. The advantages of using modern devices in rehabilitation can be seen in many areas of human performance nowadays. A common feature of gait training robots is the possibility to support (partially or totally) the body weight and the movement of patients [31]. Mobile anthropomorphic robots are examples of such modern machines which assist in the operation of human muscles and are called exoskeletons [32,33].

Furthermore, movement therapy should be stimulated by the help of psychomotor therapy, special educational methods, and therapeutic physical education that must be intentionally applied and distributed. It is a supportive method that is in parallel with pharmacotherapy and surgical approach [34,35]. This intervention supports active movement together with elements of cognitive rehabilitation and training in performing activities of daily living. Finally, we would also like to point out that it is important to motivate patients for regular exercise, whether classic or robotic because it is the lack of motivation that can lead to negative results. The reason can often be a lack of interest or non-appreciation of the regular exercise results [36]. It is an important task for physiotherapists to motivate patients towards progress.

This research has its limitation as well. We understand that in these kind of data collection there is no absolute control of the relevant variables due to the lack of randomization, so it is more vulnerable to bias. Since this is only the first study exploring the robot-assisted therapy effect in the first phase of cardiac rehabilitation in post-MI patients, the other studies should follow. Our main objective was to assess the effect by FIM score, so the other methods of evaluation are recommended for future studies as well.

## 5. Conclusions

Early rehabilitation programs for post-myocardial infarction patients might be enhanced by robotic tools such as Thera-Trainer tigo, and MOTOmed letto 2. The improvement was particularly noticeable in the case of ADLs and motor abilities, supporting the application of early robot-assisted physiotherapy. This study is the first one investigating the early impact on cardiac post-IM patients.

## Figures and Tables

**Table 1:** Characteristics of participants.

Characteristic	CG	EG	*Sig. (p-Value)*
**N** (Male/Female)	46 (25/21)	46 (25/21)	-
**Age** (60.9 ± 2.32)	60.8 ± 2.56	60.9 ± 2.08	0.96
**BMI** (32.2 ± 4.84)	31.8 ± 5.04	32.7 ± 4.63	0.21
**Diabetes duration** (7.3 ± 3.48)	7.1 ± 2.99	7.5 ± 3.93	0.63
1st day of rehabilitation program			
**Borg score** (10.3 ± 1.76)	10.5 ± 1.50	10.1 ± 1.97	0.38
**HR—rest** (76.2 ± 8.29)	77.7 ± 8.71	74.7 ± 7.66	0.14
**HR—effort** (95.0 ± 5.80)	96.0 ± 5.49	93.9 ± 5.97	0.10
7th day of the rehabilitation program			
**Borg score** (10.5 ± 1.67)	10.46 ± 1.70	10.59 ± 1.65	0.73
**HR—rest** (75.0 ± 7.73)	75.8 ± 7.64	74.15 ± 7.81	0.34
**HR—effort** (96.5 ± 5.60)	97.2 ± 5.11	95.9 ± 6.05	0.13

CG—control group, EG—experimental group, BMI—body mass index, HR—heart rate, Sig.—significance.

**Table 2:** FIM differences between the admission and 14 days of rehabilitation.

FIM Category	Admission	14 Days of Rehabilitation	Difference	Cohen’s d	Admission/14 Days of Rehabilitation *Sig. (p-Value)*
**ADL (8–56)**					
*CG*	45.11 ± 3.29	48.11 ± 3.99	2.98 ± 2.24	0.82	0.00
*EG*	45.67 ± 3.91	50.67 ± 3.49	5.02 ± 2.82	1.36	0.00
**MOTOR (5–35)**					
*CG*	16.52 ± 1.07	18.70 ± 1.44	2.17 ± 0.93	1.71	0.00
*EG*	16.61 ± 1.45	20.09 ± 1.63	3.48 ± 1.09	2.25	0.00
**SOCIAL (5–35)**					
*CG*	30.09 ± 2.31	31.38 ± 1.96	1.28 ± 1.36	0.60	0.00
*EG*	30.02 ± 2.22	31.07 ± 2.21	1.04 ± 1.03	0.47	0.00
**TOTAL SCORE (18–126)**					
*CG*	91.72 ± 4.65	98.17 ± 4.82	6.46 ± 3.17	1.36	0.00
*EG*	92.30 ± 5.07	101.83 ± 4.91	9.52 ± 3.06	1.91	0.00

FIM—functional independence measure, ADL—activities of daily living, CG—control group, EG—experimental group, Sd—standard deviation, Sig.—significance.

**Table 3:** Time * group analysis of FIM indicators.

FIM Category	Type III Sum of Squares	df	Mean Square	F	*Sig. (p-Value)*
**ADL**					
Time * Group	46.00	1	46.00	13.99	0.00 *
**Motor**					
Time * Group	19.57	1	19.57	38.24	0.00 *
**Social**					
Time * Group	0.66	1	0.66	0.90	**0.35**

FIM—functional independence measure, ADL—activities of daily living, df—degrees of freedom, F—variation between sample means, Sig.—significance.

## Data Availability

The data presented in this study are available upon request from the corresponding author.
